# Enrichment of T-cell proliferation and memory gene signatures of CD79A/CD40 costimulatory domain potentiates CD19CAR-T cell functions

**DOI:** 10.3389/fimmu.2022.1064339

**Published:** 2022-11-24

**Authors:** Socheatraksmey Ung, Pongsakorn Choochuen, Wannakorn Khopanlert, Kajornkiat Maneechai, Surasak Sangkhathat, Seitaro Terakura, Jakrawadee Julamanee

**Affiliations:** ^1^ Stem Cell Laboratory, Hematology Unit, Division of Internal Medicine, Faculty of Medicine, Prince of Songkla University, Hat Yai, Songkhla, Thailand; ^2^ Department of Biomedical Sciences and Biomedical Engineering, Faculty of Medicine, Prince of Songkla University, Hat Yai, Songkhla, Thailand; ^3^ Translational Medicine Research Center, Faculty of Medicine, Prince of Songkla University, Hat Yai, Songkhla, Thailand; ^4^ Department of Hematology and Oncology, Nagoya University Graduate School of Medicine, Nagoya, Japan

**Keywords:** gene expression profiling, CAR-T cell, CD79A/CD40, costimulatory domain, CD19

## Abstract

CD19 chimeric antigen receptor (CAR) T-cells have demonstrated remarkable outcomes in B-cell malignancies. Recently, the novel CD19CAR-T cells incorporated with B-cell costimulatory molecules of CD79A/CD40 demonstrated superior antitumor activity in the B-cell lymphoma model compared with CD28 or 4-1BB. Here, we investigated the intrinsic transcriptional gene underlying the functional advantage of CD19.79A.40z CAR-T cells following CD19 antigen exposure using transcriptome analysis compared to CD28 or 4-1BB. Notably, CD19.79A.40z CAR-T cells up-regulated genes involved in T-cell activation, T-cell proliferation, and NF-κB signaling, whereas down-regulated genes associated with T-cell exhaustion and apoptosis. Interestingly, CD19.79A.40z CAR- and CD19.BBz CAR-T cells were enriched in almost similar pathways. Furthermore, gene set enrichment analysis demonstrated the enrichment of genes, which were previously identified to correlate with T-cell proliferation, interferon signaling pathway, and naïve and memory T-cell signatures, and down-regulated T-cell exhaustion genes in CD79A/CD40, compared with the T-cell costimulatory domain. The CD19.79A.40z CAR-T cells also up-regulated genes related to glycolysis and fatty acid metabolism, which are necessary to drive T-cell proliferation and differentiation compared with conventional CD19CAR-T cells. Our study provides a comprehensive insight into the understanding of gene signatures that potentiates the superior antitumor functions by CD19CAR-T cells incorporated with the CD79A/CD40 costimulatory domain.

## Introduction

Chimeric antigen receptor (CAR) is a synthetic receptor that targets antigens and reprograms T-cell specificity, function, and persistence (1). Engineered anti-CD19CAR-T cells demonstrate remarkable clinical efficacy against various hematologic malignancies, especially in B-cell acute lymphoblastic leukemia (B-ALL) and B-cell non-Hodgkin lymphoma (B-NHL) (2, 3). However, the loss of CAR-T cell engraftment or escape variants of leukemia blasts leads to disease relapses in almost half of the patients (4). Thus, the persistence of CAR-T cells plays a crucial role in exhibiting the success of this adoptive cell treatment approach.

Previous studies demonstrated that CD19CAR incorporated with the 4-1BB enhanced CAR-T cell persistence more than incorporating the CD28 costimulatory domain (5, 6). The mechanisms by which CD28 or 4-1BB costimulated CD19CAR-T cells mediated this phenomenon have been widely investigated. Long and colleagues identified the molecular pathways that contributed to the ameliorating effect of the 4-1BB signal on CAR-T cell exhaustion (6). Another factor is distinct endogenous T-cell signaling in which CD28 mainly activates the PI3K-Akt pathway that contributes to the increment of glucose metabolism and glycolysis (7). In contrast, recent studies illustrated that the non-canonical NF-κB activated by 4-1BB costimulatory domain accelerated the CAR-T cell survival function (8, 9). In addition, 4-1BB in CAR-T promoted CD8^+^ central memory T-cells that enhanced respiratory capacity, fatty acid oxidation, and mitochondrial biogenesis (10).

Besides the T-cell costimulatory domain, B-cell-derived signaling domains have been studied to enhance CAR-T cell functions. CAR-T cell that incorporated the MyD88/CD40 costimulatory domain demonstrated greater T-cell proliferation and antitumor activity in a preclinical model (11). The additional transcriptomic analysis revealed that MyD88/CD40 promoted the expression of transcription factors associated with a less differentiated state of T-cell compared with T-cell-derived costimulatory domains (12). Recently, a study by Julamanee and colleagues developed an innovative CD19CAR structure using a novel composite of B-cell signaling moiety, CD79A/CD40 (CD19.79A.40z), to enhance both canonical and non-canonical NF-κB signaling to synergize with T-cell signaling and improve CAR-T cell function. CD19.79A.40z CAR-T cells enhanced NF-κB and nuclear factor of activated T-cell (NFAT) signaling after being stimulated with CD19 antigen. Moreover, CD19.79A.40z CAR-T cells exhibited superior antitumor activity and CAR-T cell proliferation in both B-ALL and B-NHL murine models compared with CD19CAR-T cells incorporated with either CD28 or 4-1BB (13).

To extend the knowledge of the intrinsic transcriptional gene underlying the B-cell-derived costimulatory domain, CD79A/CD40 of CD19CAR-T cell response, we investigated the differentially expressed genes (DEGs) using transcriptome analysis compared to the conventional CD28 or 4-1BB costimulated CD19CAR-T cell. Pathway enrichment analysis illustrated that CD19.79A.40z CAR-T cells up-regulated genes correlated with T-cell activation, proliferation, positive regulation of interferon production, and the NF-κB signaling pathway, and down-regulated genes mediating apoptosis and programmed cell death. Notably, a gene set enrichment analysis (GSEA) revealed that CD19.79A.40z CAR-T cells were enriched in glycolysis, fatty acid metabolism, and naïve and memory-related genes compared with conventional CAR-T cells.

## Methods

### Cell lines

The Nalm6, K562, and K562 cell lines that are genetically engineered to express CD19 (CD19-K562) are maintained in our laboratory and were used for CAR-T cell functional assays. Nalm6 cells tagged with firefly luciferase (FFluc) and enhanced with green fluorescent protein, which was established elsewhere (13), were used for the cytotoxic assay. The CD19^+^ EBV-transformed lymphoblastoid cell line (EBV-LCL) was used as a source of feeder cells for T-cell culture (14). Cell lines were cultured and maintained in RPMI-1640 containing 10% fetal bovine serum, 1% penicillin/streptomycin, and 1% L-glutamine, and incubated at 37°C in a humidified atmosphere containing 5% CO_2_.

### Human subjects

The research protocols were approved by the Human Research Ethics Committee of the Faculty of Medicine, Prince of Songkla University, Thailand (REC.64-415-14-1). Peripheral blood mononuclear cells (PBMCs) were obtained from healthy volunteer donors. Written informed consent was obtained from each donor in accordance with the Declaration of Helsinki.

### CD19CAR structures and viral vector construction

The third-generation CD19CAR structure that included the anti-CD19 single chain variable fragment (scFv)-IgG4/hinge-CD28 transmembrane domain (TM)-CD79A/CD40 intracellular domain (IC)-CD3ζ IC followed by the self-cleaving T2A sequence and a truncated version of epidermal growth factor receptor (tEGFR), which was previously established (13), was used as a template to generate a second-generation CD19CAR structure. The construct was designed to include a transduction and selection marker downstream of a T2A sequence that consisted of a truncated version of the tEGFR lacking the EGF binding and intracellular signaling domains. The T-cell costimulatory receptors CD28 and 4-1BB were generated by overlap polymerase chain reaction and assembled using NEBuider^®^ HIFi DNA Assembly Cloning Kit (New England BioLabs, Ipswich, MA, USA) to delete or insert the pre-designed costimulatory gene into the CD19CAR structure backbone. The CAR genes were then ligated into the LZRS-pBMN-Z vector and further verified by direct sequencing. Next, the CAR genes were transfected into the Phoenix-Ampho (Orbigen, San Diego, CA, USA) retroviral packaging cells to make gamma retroviral supernatants.

### Generation, expansion, and selection of CD19CAR-T cells

PBMCs were isolated from the whole blood of healthy volunteers using Lymphoprep™ (StemCell Technologies Inc., Canada) density-gradient centrifugation. CD3^+^ cells were purified with immunomagnetic beads (Miltenyi Biotec, Bergisch Gladbach, Germany) and activated using anti-CD3/CD28 beads (Invitrogen, Carlsbad, CA, USA). CD3^+^ cells were then cultured in RPMI-1640 medium containing 10% human serum, 0.8mM L-glutamine, 1% penicillin/streptomycin, and 0.5 μM 2-mercaptoethanol (cytotoxic T-cell medium; CTL), which was supplemented with 50 IU/ml of recombinant human interleukin-2 (IL-2). The activated CD3^+^ cells are retrovirally transduced on day 3 using recombinant human retronectin fragment-coated plates (Retronectin, Takara Bio, Otsu, Japan) and centrifuged at 2100 rpm for 1 h at 32°C. On day 7, CAR^+^ T-cells were purified using biotin-conjugated anti-EGFR monoclonal antibody and counterstained with anti-biotin beads (Miltenyi Biotec). The enriched CAR^+^ T-cells were further expanded by culturing with γ-irradiated EBV-LCL feeder cells at a responder:stimulator ratio of 1:7 for 10 days until a sufficient number of cells for downstream experiments was obtained (14).

### CAR-T cell proliferation assay

Untransduced- or CD19CAR-T cells were stimulated once with the γ-irradiated CD19-K562 cell line at an target to target (E:T) ratio of 1:1 and cultured in medium with or without exogenous IL-2 supplementation for 10 days. T-cell proliferation was measured by counting viable cells using trypan blue at indicated time points.

### Prolonged co-culture assay

Untransduced- or CD19CAR-T cells were co-cultured with Nalm6-FFluc at various E:T ratios (1:1, 1:8, 1:16) without IL-2 supplementation. The percentage of residual target cells was assessed using the flow cytometry technique at various time points up to nine days.

### Cytokine secretion assay

Untransduced- or CD19CAR-T cells were stimulated with the γ-irradiated CD19-K562 cell line at an E:T ratio of 1:1 and cultured in medium without IL-2 supplementation for 16 hours. The cell culture supernatants were collected, and the IL-2, interferon-γ (IFN-γ), and tumor necrosis factor-α (TNF-α) concentrations were measured using sandwich ELISA (BD Biosciences, San Jose, CA, USA).

### CAR-T cell immunophenotypes

Untransduced- or CD19CAR-T cells were cultured with the γ-irradiated CD19-K562 cell line in a 1:1 ratio for seven days without IL-2 supplementation. The T-cells were stained with monoclonal antibodies conjugated with fluorophores: CD3, CD8, CD45RA, CD62L (BD Biosciences), PD-1, LAG-3, CTLA-4, and TIM-3 (BioLegend, San Diego, CA, USA). The tEGFR^+^ cells were stained with biotinylated anti-EGFR antibody (R&D Systems, Bio-Techne, MN, USA) and counterstained with streptavidin-phycoerythrin (BD Biosciences). All samples were analyzed by a CytoFlex S flow cytometer machine (Beckman Coulter Inc, CA, USA) and data were analyzed using FlowJo software (Tree Star).

### RNA extraction and RNA sequencing

mRNA was extracted from cells using RNA Blood Mini Kit (QIAGEN, Germany) according to the manufacturer’s instructions. RNA concentration and purity were measured by a NanoDrop™ spectrophotometer (Thermo Fisher Scientific). The RNA integrity number (RIN) was assessed using the Agilent Technologies 2100 Bioanalyzer. Total RNA 100–200 ng was used to undergo polyA selection and TruSeq RNA library preparation according to the manufacturer’s instructions (TruSeq Stranded mRNA LT Kit; Illumina). The samples were barcoded and run on a Hiseq 4000 (Illumina).

### Differential gene expression analysis

A summary of the RNA analysis pipeline used in this study is shown in **Figure S2A**. A total of nine RNA samples (RIN score above 7) from three healthy donors were RNA-seq. The quality control of raw sequence read data in the FASTQ file from RNA-seq was assessed using the FastQC tool before further analysis to avoid inaccurate results. The Trimmomatic tool was used to trim the adapter sequences or other contaminating sequences. Short sequence reads were mapped to the reference genome from GENCODE (GRCh38) to identify their genomics position using HISAT2. The expression levels of each gene were estimated by counting the number of reads aligned to each full-length transcript. The expression count matrix was then computed from the mapped reads using HTSeq. The EdgeR program, a Bioconductor package, was used to perform the DEG analysis between the three groups: CD19.79A.40z vs. CD19.28z, CD19.79A.40z vs. CD19.BBz, and CD19.28z vs. CD19.BBz. The false discovery rate (FDR) was calculated using the Benjamini-Hochberg method to adjust the p-value. The DEGs were selected at FDR ≤ 0.05 and fold change ≥ 1.5-fold difference. Volcano plots and heatmaps of the DEGs were generated using the EnhancedVolcano package and Pheatmap package, respectively, in R version 4.1.2.

### Pathway enrichment analysis

To identify regulation of functionally related gene ontology (GO) terms and enriched pathways, we performed both ranked gene list analyses on the whole gene expression level using GSEA software version 4.2.3 (15) and the DEG list was separately analyzed for up- and down-regulated genes using g:Profiler version e105_eg52_p16_e84549f (16). The input of GSEA was a ranked list of all available genes with normalized counts generated in EdgeR without using a cut-off in each comparison group. The reference gene sets used from the Molecular Signatures Database (MsigDB) were h.all.v7.5.1.symbols (hallmark), c2.cp.reactome.v7.5.1.symbols (curated), c5.all.v7.5.1.symbols (gene ontology), and c7.all.v7.5.1.symbols (immunologic signature).

GSEA assesses genes from the top to the bottom of the ranked list to produce an enrichment score (ES) for a pathway, which increases the ES if a gene is part of the pathway and decreases the score otherwise. Enrichment in the top and bottom ranking genes is amplified, whereas enrichment in genes with more moderate ranks is not amplified. The normalized enrichment score (NES) reflects the enrichment of the pathway in the list and is derived as the maximum value of the running sum normalized relative to pathway size. Enrichment at the top and bottom of the list is represented by positive and negative NES values, respectively. A nominal p-value ≤ 0.05 and FDR q-value ≤ 0.25 were considered statistically significant in the enrichment plots (15).

The functional enrichment analysis of DEGs in g:Profiler was performed using the function g:Gost functional profiling to map the input genes list and identify the statistically significant enriched terms on gene ontology-biological process (GO-BP) and REACTOME databases. The significance threshold of 0.05 was applied using the g:SCS multiple testing correction method.

### Statistical analysis

Statistical analyses of *in-vitro* experiments were performed using GraphPad Prism version 9.4.0 (GraphPad Software, La Jolla, CA, USA). All of the experimental data are presented as mean ± SEM. The differences among results were evaluated with one-way ANOVA or two-way ANOVA with Bonferroni’s or Tukey’s post-test correction when appropriate. Differences were considered statistically significant when p ≤ 0.05.

## Results

### CD79A/CD40 enhanced CD19CAR-T cell proliferation after CD19 antigen stimulation

**Figure 1:**
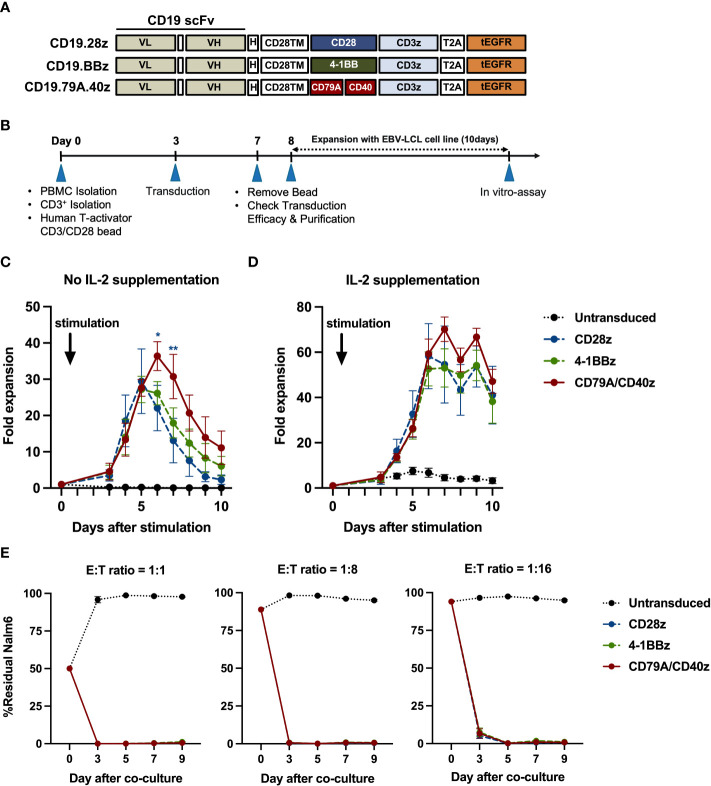
CD19CAR-T cell structure and CD19CAR-T cell response upon CD19^+^ target cell stimulation in an *in vitro* assay. **(A)** Schematic of CD19CAR-T cell structures. Each costimulatory domain either CD28 (CD28z), 4-1BB (BBz), or CD79A/CD40 (CD79A.40z) was fused into anti-CD19 scFv-H-CD28TM followed by CD3ζ and tEGFR. scFv, single chain variable fragment; VL, light-chain variable fragment; VH, heavy chain variable fragment; H, short 12 amino acid of IgG4 Fc-derived spacer of hinge; TM, transmembrane domain; tEGFR, truncated EGFR. **(B)** Experimental schematic of CD19CAR-T cell generation and functional analysis. EBV-LCL, EBV-transformed lymphoblastoid cell line. **(C, D)** T-cell proliferation assay. Untransduced or CD19CAR-T cells were stimulated with γ-irradiated CD19-K562 cell line in 1:1 ratio for 10 days and cultured without **(C)** IL-2 supplementation or with **(D)** IL-2 supplementation (50 IU/ml). T-cell proliferation were measured by counting viable cells. Arrows mark the day of CD19-K562 cell stimulation. **(E)** Prolonged co-culture assay. Untransduced or CD19CAR-T cells were co-cultured with Nalm6-FFluc at E:T ratios of 1:1 (left), 1:8 (middle), and 1:16 (right) for a total of nine days without exogenous IL-2. The remaining Nalm6-FF were assessed by flow cytometry at the indicated time points. All data were pooled from three different donors and are presented as mean ± SEM. 2-way ANOVA for **(C, D)**; *p < 0.05, **p < 0.01 (CD19.79A.40zCAR- vs. CD19.28z CAR-T cells); One-way ANOVA for **(E)**.

**Figure 2:**
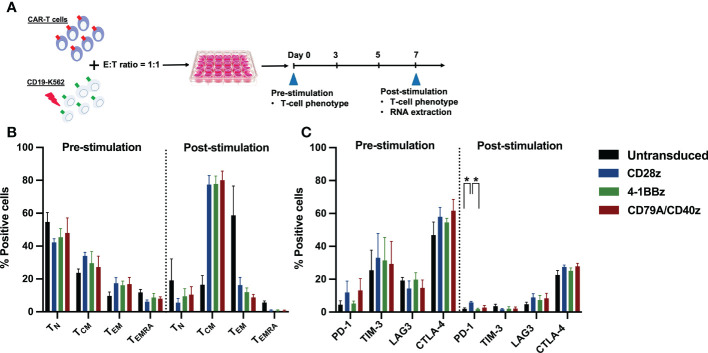
CAR-T cell immunophenotype assays. **(A)** Experimental schematic of antigen stimulation and CD19CAR-T cell phenotype assays. CD19CAR-T cells were stimulated with γ-irradiated CD19-K562 cell line in a 1:1 ratio for seven days and cultured without IL-2 supplementation. Then, the CD19CAR-T cell proliferation, T-cell differentiation, and exhaustion phenotypes were assessed before and after stimulation on day 0 and day 7, respectively. The remaining CD19CAR-T cells 3–6 x 10^6^ cells were harvested at post stimulation on day 7 and extracted for RNA for further RNA sequencing. **(B)** The percentages of T-cell differentiation subsets were determined by CD62L^+^ CD45RA^+^ naïve T (T_N_), CD62L^+^ CD45RA^-^ central memory T (T_CM_), CD62L^-^ CD45RA^-^ effector memory T (T_EM_), and CD62L^-^ CD45RA^+^ effector memory re-expressing CD45RA T (T_EMRA_) cells before and after stimulation. **(C)** The percentage of T-cell exhaustion was determined by exhausted T-cell markers PD-1^+^, TIM-3^+^, LAG-3^+^, and CTLA-4^+^ before and after stimulation. Data were pooled from three different donors and are shown as mean ± SEM; One-way ANOVA for **(B, C)**; *p < 0.05.

To confirm whether each CAR-T cell structure contained proper functions, we developed a CD19CAR structure that incorporated either the CD28, 4-1BB, or CD79A/CD40 gene (**Figure 1A**). CD19CAR-T cells were successfully generated and expanded with feeder cells before an *in vitro* assay (**Figures 1B**, **S1A**). We first examined T-cell proliferation after stimulation with the CD19 antigen. The CD19.79A.40z CAR-T cells exhibited superior CAR-T cell proliferation regardless of IL-2 supplementation (**Figures 1C, D**). To assess cytotoxicity, we performed a prolonged co-culture assay. The T-cells were cultured with CD19^+^ target cells at various effector to target cell ratios without IL-2 supplementation. After three days of culture, all CD19CAR-T cells could suppress tumor cell growth until the last day of the co-culture assay (**Figure 1E**). In terms of cytokine secretion, we observed similar amounts of IFN-γ, IL-2, and TNF-α in the culture medium after stimulation with target cells in the ratio of 1:1 for 16 hours (**Figure S1B**). To investigate the T-cell immunophenotype and the intrinsic transcriptional gene following target antigen exposure, we stimulated CAR-T cells with CD19 antigen and cultured them for seven days without exogenous IL-2. The T-cells were assessed for T-cell expansion and immunophenotypes for T-cell subsets and exhaustion markers before and after stimulation (**Figure 2A**). Higher T-cell proliferation was again observed in CD19.79A.40z CAR-T cells compared with CD19.28z CAR-T cells (**Figure S1C**). Regarding T-cell subsets, CAR-T cells predominantly expressed naïve and central memory phenotypes at pre-stimulation and mostly differentiated into central memory T-cells after stimulation without any differences among the CAR constructs (**Figure 2B**). Higher expressions of the T-cell exhaustion phenotypes, which included PD-1, TIM-3, LAG-3, and CTLA-4, were observed at pre-stimulation compared with the post-stimulation phase among the CD19CAR constructs. It was observed that only CD19.28z CAR-T cells highly expressed PD-1 after stimulation compared with the others (**Figure 2C**). In addition, similar overexpressions of TIM-3 and CTLA-4 were observed among the CAR structures at baseline and only CTLA-4 after stimulation. From these results, we confirmed a higher CAR-T cell proliferation of CD19CAR that incorporated the CD79A/CD40 costimulatory domain compared with the T-cell-derived costimulatory domain.

### Distinctive gene expressions of CD19.79A.40z CAR-T cells after stimulation with target cells

**Figure 3:**
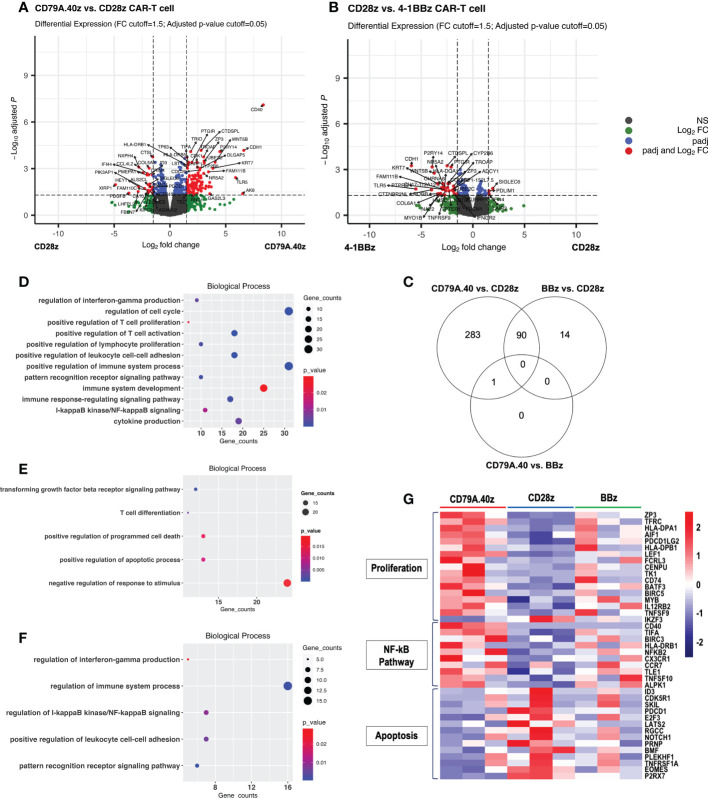
Differential gene expression profiling analysis of CD19CAR-T cell. **(A, B)** Volcano plots of DEGs between **(A)** CD19.28z CAR-T cells vs. CD19.79A.40z CAR-T cells and **(B)**, CD19.BBz CAR-T cells vs. CD19.28z CAR-T cells. Visualization of DEGs in volcano plots. Up-regulated- and down-regulated genes with FDR < 0.05 are marked in red. DEGs were selected with thresholds of false discovery rates (FDR) ≤ 0.05 and ≥ 1.5-fold difference. **(C)** Venn diagram illustrating the number of overlapped DEGs between each comparison: CD19.79A.40z CAR-T cells vs. CD19.28z CAR-T cells, CD19.79A.40z CAR-T cells vs. CD19.BBz CAR-T cells, and CD19.BBz CAR-T cells vs. CD19.28z CAR-T cells. **(D–F)** Gene ontology-biological processes (GO-BP) of up- **(D)** and down-regulated DEGs **(E)** in CD19.79A.40z CAR-T cell compared to CD19.28z CAR-T cells. **(F)** The up-regulated DEGs in CD19.BBz CAR-T cells compared to CD19.28z CAR-T cells. **(G)**. Heatmap of normalized counts, under the indicated CD19CAR-T cells in three major categories related to proliferation genes, NF-κB pathway genes, and apoptosis genes. Gene names are listed on the right side and each CAR structure is marked at the top of the colored map. The color bar indicates the normalized counts values.

To further investigate the transcriptomic differences among CAR structures, RNA was extracted from the CD19CAR-T cells after stimulation with target cells for seven days (**Figure 2A**). The DEGs of the three groups were assessed and compared: CD19.79A.40z CAR-T cells vs. CD19.28z CAR-T cells; CD19.79A.40z CAR-T cells vs. CD19.BBz CAR-T cells; and CD19.28z CAR-T cells vs. CD19.BBz CAR-T cells. The distributions of all DEGs obtained from each comparison and the gene names of the most significant and highest expression changes from the DEG list are shown in volcano plots (**Figures 3A, B**) with statistical significance of DEG data (adjusted p-value) versus the magnitude of the expression change (log2 fold change). According to the DEG analysis using EdgeR, the CD19.79A.40z CAR-T cells resulted in significant changes in the expression of 374 genes compared with the CD19.28z CAR-T cells: 232 genes were up-regulated and 142 genes were down-regulated (**Figures 3A, C**). Notably, we observed the up-regulation of T-cell activation genes included major histocompatibility complex class II genes (*HLA-DP, HLA-DR*, and *HLA-DM*), *CCR7, TFRC, LEF1, MYB, ZP3, CD74*, and *MAP3K8*, while down-regulation of T-cell exhaustion and apoptosis genes included *ID3*, *PDCD1*, and *EOMES* in CD19.79A.40z CAR-T cells (**Figure 3A**). Surprisingly, only one up-regulated gene, CD40, was identified among the CD19.79A.40z CAR- and CD19.BBz CAR-T cells (**Figure S2B**). Moreover, 104 DEGs were observed among the CD19.28z CAR- and CD19.BBz CAR-T cells, in which 32 DEGs were up-regulated and 72 DEGs were down-regulated in CD19.28z CAR-T cells (**Figures 3B, C**). From the DEG analysis, CD19.79A.40z CAR-T cells exhibited markedly up- and down-regulated genes compared with CD19.28z CAR-T cells after antigen stimulation. Nonetheless, we identified similar gene expression profiling among the CD19.79A.40z CAR- and CD19.BBz CAR-T cells, which was correlated to greater proliferative capacity in these two co-stimulatory domains.

### Advantage of CD19.79A.40z CAR-T cell proliferation and persistence related to various biological pathways

We next sought to define the molecular pathways contributing to the greater CAR-T cell functions. The DEGs were analyzed separately for up- and down-regulated genes in terms of gene ontology categories and enriched biological pathways. The findings obtained from the DEG data of CD19.79A.40z CAR-T cells vs. CD19.28z CAR-T cells using the g:Profiler software indicated significant categories of pathway enrichment according to the gene ontology-biological process (GO-BP) database (**Figures 3D, E**). The up-regulated DEGs in the CD19.79A.40z CAR-T cells revealed that the most significant enriched pathways were those related to positive regulation of T-cell proliferation, T-cell activation, cell-cell adhesion, IFN-γ production, cytokine production, and I-κB kinase/NF-κB signaling (**Figure 3D**). In contrast, the enriched GO-BP of down-regulated DEGs in CD19.79A.40z CAR-T cells were transforming growth factor beta receptor signaling pathway, positive regulation of apoptotic process, and programmed cell death (**Figure 3E**).

Furthermore, only up-regulated DEGs in CD19.BBz CAR-T cells compared with CD19.28z CAR-T cells were significantly enriched in GO-BP and similar to those in CD19.79A.40z CAR-T cells (**Figure 3F**). Of note, since only one DEG was identified among CD19.79A.40z CAR-T cells vs. CD19.BBz CAR-T cells, no enrichment results were obtained from the analysis. The three major DEG pathways related to T-cell proliferation, NF-κB signaling, and apoptosis are shown in the heatmap (**Figure 3G**). Significant up-regulation of key transcriptional regulators of T-cell proliferation, such as *ZP3*, *TFRC*, *HLA-DPA1*, *AIF1*, *HLA-DPB1*, *TNFSF9*, *BATF3*, *CD74*, *IL12RB2*, and *MYB* as well as NK-κB related genes including *CD40*, *BIRC3*, *NFKB2*, *CCR7*, and *TNFSF10*, was observed in CD19.79A.40z CAR-T cells compared with the others. On the other hand, CD19.79A.40z CAR-T cells also down-regulated apoptosis-related genes including *ID3*, *PDCD1*, *NOTCH1*, and *EOMES*. To conclude, the pathway enrichment analysis demonstrated that CD19.79A.40z CAR-T cells significantly up-regulated genes regulated T-cell proliferation, T-cell activation, and NF-κB signaling, whereas down-regulated genes related to apoptotic process and programmed cell death, which potentiated CAR-T cell proliferation.

### CD19.79A.40z CAR-T cells enriched in genes related to T-cell proliferation, memory signatures, and less expressed genes regulated T-cell exhaustion

**Figure 4:**
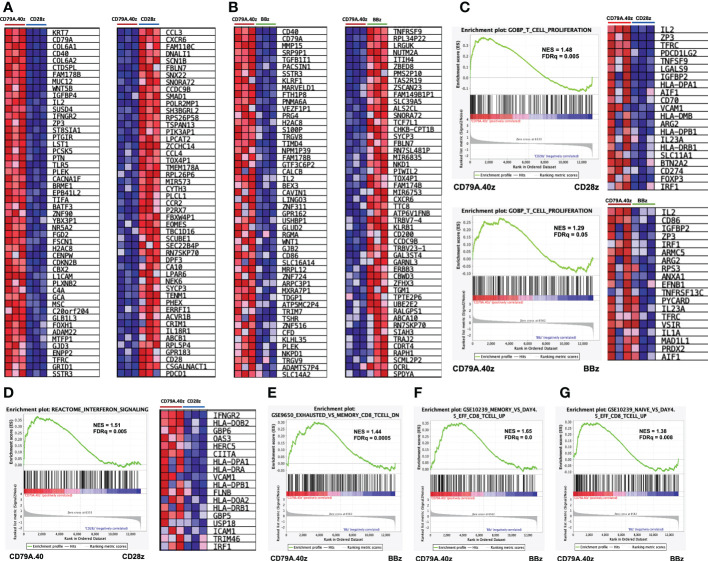
Gene set enrichment analysis of CD19.79A.40z CAR-T cells following antigen stimulation. **(A, B)** Heatmap of the top 50 up- (left) or down- (right) regulated genes differentially expressed by either **(A)** CD19.79A.40z CAR-T cells vs. CD19.28z CAR-T cells or **(B)** CD19.79A.40z CAR-T cells vs. CD19.BBz CAR-T cells. The colors reflect expression values of normalized counts, which range from red (high expression), pink (moderate expression), light blue (low expression), to dark blue (lowest expression). **(C)** Representative GSEA results of enriched GO-BP T-cell proliferation in CD79A.40z CAR-T cells vs. CD19.28z CAR-T cells (upper) and CD79A.40z CAR-T cells vs. CD19.BBz CAR-T cells (lower) with heatmap of the top up-regulated genes (right). **(D)** GSEA of significantly enriched in interferon signaling pathway of CD79A.40z CAR-T cells vs. CD19.28z CAR-T cells, using the REACTOME database with heatmap of the top up-regulated genes (right). **(E)** GSEA enriched in down-regulation of exhausted-relative to memory CD8 T-cell related gene in CD19.79A.40z CAR-T cells vs. CD19.BBz CAR-T cells. **(F, G)** GSEA up-regulated in **(F)** memory-relative to effector-related genes and **(G)** naïve-relative to effector-related genes of CD19.79A.40z CAR-T cells vs. CD19.BBz CAR-T cells. The upper part of each GSEA plot displays the enrichment score, whereas the lower part displays the ranked list metric of the gene set. The ranked gene list is shown in the middle: red indicates up-regulation; blue indicates down-regulation; and the black vertical line indicates the gene set. FDR q-value cutoff ≤ 0.25, Nominal p-value ≤ 0.05.

To interpret the genome-wide transcriptional profiles related to T-cell function, differentiation, and exhaustion of CD19CAR-T cells after antigen stimulation, we further characterized the molecular pathway using GSEA. A comparison of the top 50 gene markers of CD19.79A.40z CAR-T cells vs. CD19.28z CAR-T cells is shown in **Figure 4A**. *CD79A*, *CD40*, *IL2*, *IFNGR2*, *ZP3*, and *BATF3* were in the top 25 genes most correlated with the CD19.79A.40z CAR-T cell phenotype. In contrast, *CXCR6*, *P2RX7*, *EOMES*, *CD28*, and *PDCD1* were in the top 25 genes most correlated with CD19.28z CAR-T cells, in which higher PD-1 expression was observed in CD19.28z CAR-T cells following CD19 antigen stimulation compared with the other constructs (**Figure 2C**). The top 25 genes most correlated with the CD19.79A.40z CAR-T cell phenotype versus the top 25 genes most correlated with the CD19.BBz CAR-T cell phenotype are also illustrated in **Figure 4B**.

The GSEA demonstrated that the CD19.79A.40z CAR-T cells enriched in genes previously identified to correlate with T-cell proliferation, compared with either CD19.CD28z CAR- or CD19.BBz CAR-T cells, included *IL-2*, *ZP3*, *TFRC*, and *CD70* (**Figure 4C**). Moreover, we further examined the enrichment of ranked gene lists in interferon signaling pathway using the REACTOME database. Significant up-regulation of the gene set associated with interferon signaling was found in CD19.79A.40z CAR-T cells, such as *IFNGR2* (type II IFN-γ), *HLA-DOB2*, *GBP6*, *OAS3*, *HERC5*, *FLNB*, and *IRF1*, compared with CD19.28z CAR-T cells (**Figure 4D**). Compared with CD19.BBz CAR-T cells, CD19.79A.40z CAR-T cells down-regulated the exhausted-related genes of CD8 T-cells (**Figure 4E**). In contrast, the enrichment in gene sets associated with memory and naïve CD8 T-cell were predominantly observed in CD19.79A.40z CAR-T cells (**Figures 4F, G**).

Furthermore, we analyzed other GSEA gene sets using the hallmark collection. The CD19.79A.40z CAR-T cells were strongly related to various pathways compared with the CD19.28z CAR- or CD19.BBz CAR-T cells such as E2F target genes, Mtorc1 signaling, Myc target v1, and oxidative phosphorylation (**Figures S3A–H**). Regarding CAR-T cell metabolism, CD19.79A.40z CAR-T cells demonstrated an enrichment in genes related to both fatty acid and glycolysis metabolism compared with the others (**Figures S3I–K**). Our data suggested that CD19.79A.40z CAR-T cells responded to target cell stimulation by up-regulating genes related to T-cell activation, interferon signaling, memory-related signatures, fatty acid and glycolysis metabolism, as well as down-regulating the exhausted gene signatures.

## Discussion

The costimulatory domain significantly impacts the functions of CAR-T cells. To date, the most extensively employed costimulatory domains within CD19-targeted CARs have been CD28 and 4-1BB (17). The 4-1BB-costimulated CAR-T cells have substantially slower kinetics but have higher persistence. On the other hand, CD28-costimulated CAR-T cells are linked to more rapid T-cell proliferation and tumor eradication (18). In this study, we constructed three CD19CAR structures with different costimulatory domains (CD28, 4-1BB, or CD79A/CD40) and assessed their functions in an *in vitro* assay. CD19.79A.40z CAR-T cells showed higher proliferative capacity than CD19.28z CAR- or CD19.BBz CAR-T cells, which was consistent with a previous report (13). The strong NF-κB, NFAT, and p38 nuclear-translocating signals generated by the CD79A/CD40 costimulatory domain following CD19 antigen stimulation were proposed as the contributing factors for greater CAR-T cell proliferation and persistence. From RNA sequencing analysis, we provided informative data on gene expression profiling of CD19CAR-T cells incorporated with a B-cell costimulatory domain in response to short-term antigen stimulation. The pathway enrichment analysis suggested up-regulated DEGs in T-cell proliferation, T-cell activation, cell cycle regulation, regulation of interferon production, and NF-κB pathway in CD19.79A.40z CAR-T cells compared with CD19.28z CAR-T cells. Interestingly, we found that CD19.79A.40z CAR- and CD19.BBz CAR-T cells were enriched in almost similar pathways, which translated into a similar advantage in T-cell proliferation and persistence of these costimulatory structures.

T-cell differentiation subsets of all CAR-T constructs predominantly expressed naïve and central memory phenotypes at pre-stimulation and most differentiated into central memory T-cells after stimulation according to their self-renewal capacity compared with effector T-cells that were undetectable at the end of culture. Higher T-cell exhaustion was demonstrated by higher PD-1 expression in CD19.28z CAR-T cells following CD19 antigen stimulation compared with the other constructs which correlated with the up-regulated *PDCD1* gene in CD19.28z CAR-T cells from RNA sequencing analysis.

The previous study by Long and colleagues investigated the molecular pathways contributing to the ameliorating effect of the 4-1BB signal on CAR-T cell exhaustion compared to CD28. They did not demonstrate any differences in transcription factors associated with memory generation between 4-1BB and CD28 in the CD19CAR-T cell model; however, differences appeared in the GD2CAR-T cell model. The GD2.28z CAR-T cells showed higher expression of genes encoded by inhibitory receptors or transcription factors such as *LAG-3*, *TIM-3*, *TBX21*, and *EOMES*. On the other hand, GD2.BBz CAR-T cells highly expressed genes encoded by transcription factors associated with memory such as *KLF6*, *JUN*, and *JUNB* (6). In this study, CD19.79A.40z CAR-T cells enriched in genes associated with T-cell proliferation, NK-κB signaling, and naïve and memory signatures such as *ZP3*, *IFNGR2*, *BATF3*, *TNFSF9*, *BATF3*, *CD74*, *IL12RB2*, *MYBBIRC3*, *NFKB2*, *CCR7*, and *TNFSF10*. Furthermore, less expressed genes related to apoptosis and T-cell exhaustion included *CXCR6*, *P2RX7*, *ID3*, *NOTCH1*, *EOMES*, and *PDCD1*. Similar results from a previous study by Prinzing et al. reported a transcriptomic analysis of B-cell costimulation of MyD88/CD40 in CAR-T cells in solid tumor models. Higher levels of *MYB* and *FOXM1*, the key cell regulators, and low levels of *TBET* and *BLIMP1* were identified, which promoted terminal T-cell differentiation compared with CD28- or 4-1BB-endowed CAR-T cells. They concluded that MyD88/CD40 costimulation is a less differentiated CAR-T phenotype, which translates into greater proliferative capacity and persistence (12). These findings highlighted the less-differentiated T-cell subsets and memory characteristics of the CAR-T cells that incorporated the B-cell-derived costimulatory domain, which translated into better T-cell persistence and enhanced antitumor activity (12, 13, 19, 20).

Among the DEGs, we discovered an up-regulation of the *BATF3* gene in CD79A/CD40 over the others, which enhanced T-cell proliferative capacity and memory phenotypes. A recent study by Ataide et al. demonstrated that long-lasting *BATF3* expression in T-cells promoted survival and transition to a memory T-cell phenotype. *BATF3* also regulated T-cell apoptosis and longevity *via* the pro-apoptotic factor *BIM* (21). In addition, CD79A/CD40 also significantly down-regulated the Ikaros Family Zinc Finger Protein 3, *IKZF3* gene. Immune cell development and cytokine signaling are two main functions of the IKZF family members that have been thoroughly established (22). A recent study indicated that the knock-out *IKZF3* gene (*IKZF3* KO) enhanced proliferation and could potentiate the killing effect of CAR-T cells in solid tumor cells *in vitro* and xenograft models by increasing the expression of genes mediating cytokine signaling and cytotoxicity (23).

Concomitantly, GSEA also demonstrated the enrichment genes in Mtorc1 signaling and Myc target v1, which are the pathways involved in cell cycle, proliferation, differentiation, and survival (24, 25). The metabolic pathway of the CD19.79A.40z CAR-T cells was demonstrated by enrichment of both glycolysis and fatty acid metabolism compared with the others, which correlated with the high NF-κB and NFAT signaling following CD19 antigen stimulation in the recent study (13). Previous studies demonstrated that CD28-costimulated CD19CAR-T cells enhanced glycolytic metabolism and induced an effector memory phenotype. In contrast, 4-1BB-based CD19CAR-T cells were found to depend on fatty acid metabolism and induced a central memory phenotype (10, 26). The underlying metabolic pathway is closely linked to T-cell activation and proliferation (27). The activation of T-cells requires metabolic programming to support the proliferation and differentiation of naïve T-cells upon antigen recognition (7, 28). Following T-cell activation, the expression of glycolysis and glutaminolysis-related genes are up-regulated to produce extracellular nutrients including glucose, glutamine, and amino acids (28–30). Fatty acid synthesis then proceeds to support T-cell proliferation (27).

Besides CD19CAR-T cells, we tested CD79A/CD40 costimulatory domain in other CAR models: CD20CAR-T and CD37CAR-T cells. CD20CAR incorporated with CD79A/CD40 or 4-1BB costimulatory domain showed higher proliferative capacity compared with CD28 after stimulation with CD20-K562 cells. In contrast, we did not observe any difference in CAR-T cell proliferation among costimulatory domains in CD37CAR-T cells after stimulation with CD37 positive antigen. There was no significant difference among the costimulatory domains in both CAR-T cell models regarding cytokine secretion and cytotoxicity. The inconsistent results of CD79A/CD40 costimulatory domain among CD19CAR-T cell and 20CAR-T or CD37CAR-T cell models were possibly caused from multifactorial factors including the differences in antigen binding affinity, the flexibility, and extracellular protein folding of antiCD20 or antiCD37 scFv compared with antiCD19scFv (FMC63 clone). Further studies are needed to confirm the efficacy of the novel B-cell signaling molecules in other types of CAR-T cells. Regarding limitation of study, we did not assess the protein expressions to confirm the RNA sequencing results, which was out of our scope and the further studies may be needed.

In conclusion, this study provided comprehensive gene expression profiling of CD19CAR-T cells incorporated with the B-cell-derived costimulatory domain, CD79A/CD40, which significantly up-regulated genes related to T-cell activation, proliferation, interferon production, NF-κB signaling, memory signatures, and down-regulated apoptotic and T-cell exhaustion genes compared with CD28 or 4-1BB. In addition, both glycolysis and fatty acid metabolism pathways, which are necessary to drive T-cell proliferation and differentiation, were also highly enriched in CD79A/CD40 compared with conventional costimulatory domains.

## Data availability statement

The datasets presented in this study can be found in online repositories. The names of the repository/repositories and accession number(s) can be found below: NCBI under accession ID: PRJNA896859.

## Ethics statement

The studies involving human participants were reviewed and approved by The Human Research Ethics Committee of the Faculty of Medicine, Prince of Songkla University, Thailand (REC.64-415-14-1). The patients/participants provided their written informed consent to participate in this study.

## Author contributions

Conception and design; SU, ST, and JJ. Development of methodology; SU, PC, and JJ. Acquisition of data; SU, PC, WK, KM, SS, ST, and JJ. Analysis and interpretation of data (e.g., statistical analysis, computational analysis); SU, PC, and JJ. Writing, review, and/or revision of the manuscript; SU, PC, WK, KM, SS, ST, and JJ. Administrative, technical, or material support (e.g., reporting or organizing data, constructing databases); SU, PC, and JJ. Study supervision; SS, ST, and JJ. All authors contributed to the article and approved the submitted version.

## Funding

This work was supported by the National Science, Research and Innovation Fund (NSRF) and Prince of Songkla University (Grant No. MED6505092S to JJ), the Higher Education Research Promotion, and the Thailand Scholarships of the Higher Education Commission (Grant No.1272/2552 to SU).

## Acknowledgments

The authors would like to thank the Stem Cell Laboratory, Department of Biomedical Science and Bioengineering, and Translational Medicine Research Center, Faculty of Medicine, Prince of Songkla University for the technical assistance.

## Conflict of interest

The authors declare that the research was conducted in the absence of any commercial or financial relationships that could be construed as a potential conflict of interest.

## Publisher’s note

All claims expressed in this article are solely those of the authors and do not necessarily represent those of their affiliated organizations, or those of the publisher, the editors and the reviewers. Any product that may be evaluated in this article, or claim that may be made by its manufacturer, is not guaranteed or endorsed by the publisher.

## Supplementary material

Supplementary Figure 1
**(A)** Transduction efficacy and purification of CD19CAR-T cells. **(B)** Concentration of IFN-γ, IL-2, and TNF-α secretion. T-cells were stimulated with γ-irradiated CD19-K562 cell line in a 1:1 ratio for 16 hours, and culture supernatants were analyzed using an ELISA. **(C)** Fold expansion of T-cells following γ-irradiated CD19-K562 cell line stimulation in a 1:1 ratio for seven days and cultured without IL-2 supplementation. Arrows mark the day of CD19-K562 cell stimulation. All data were pooled from three different donors and are shown as mean ± SEM. One-way ANOVA for **(A)** and **(B)**; 2-way ANOVA for **(C)**; ***p < 0.001 (CD19.79A.40zCAR- vs. CD19.28z CAR-T cells).

Supplementary Figure 2
**(A)** RNA-analysis pipeline. **(B)** Volcano plots of DEGs between CD19.79A.40z CAR-T cells vs. CD19.BBz CAR-T cells. Visualization of DEGs in volcano plots. Up-regulated- and down-regulated genes with FDR < 0.05 are marked in red. DEGs were selected with thresholds of false discovery rate (FDR) ≤ 0.05 and ≥ 1.5-fold difference.

Supplementary Figure 3
**(A–D, K)** GSEA indicated the gene enrichments in CD19.79A.40z CAR-T cells vs. CD19.28z CAR-T cells in the following hallmarks; **(A)** E2F target, **(B)** Mtorc1 signaling, **(C)** Myc target v1, **(D)** oxidative phosphorylation, and **(K)** glycolysis. **(E–J)** GSEA indicated gene enrichment in CD19.79A.40z CAR-T cells vs. CD19.BBz CAR-T cells in the following hallmarks; **(E)** E2F target, **(F)** Mtorc1 signaling, **(G)** Myc target v1, **(H)** oxidative phosphorylation, **(I)** fatty acid metabolism, and **(J)** glycolysis. The upper part of each plot displays the enrichment score, whereas the lower part displays the ranked list metric of the gene set. The ranked gene list is shown in the middle: red indicates up-regulation; blue indicates down-regulation; and the black vertical line indicates the gene set. FDR q-value cutoff ≤ 0.25, nominal p-value ≤ 0.05.
