# Supplementary figures and images for: Enrichment of T-cell proliferation and memory gene signatures of CD79A/CD40 costimulatory domain potentiates CD19CAR-T cell functions
Source: Front Immunol. 2022 Nov 24;13:1064339. doi: 10.3389/fimmu.2022.1064339 (PMC9729744; doi:10.3389/fimmu.2022.1064339)

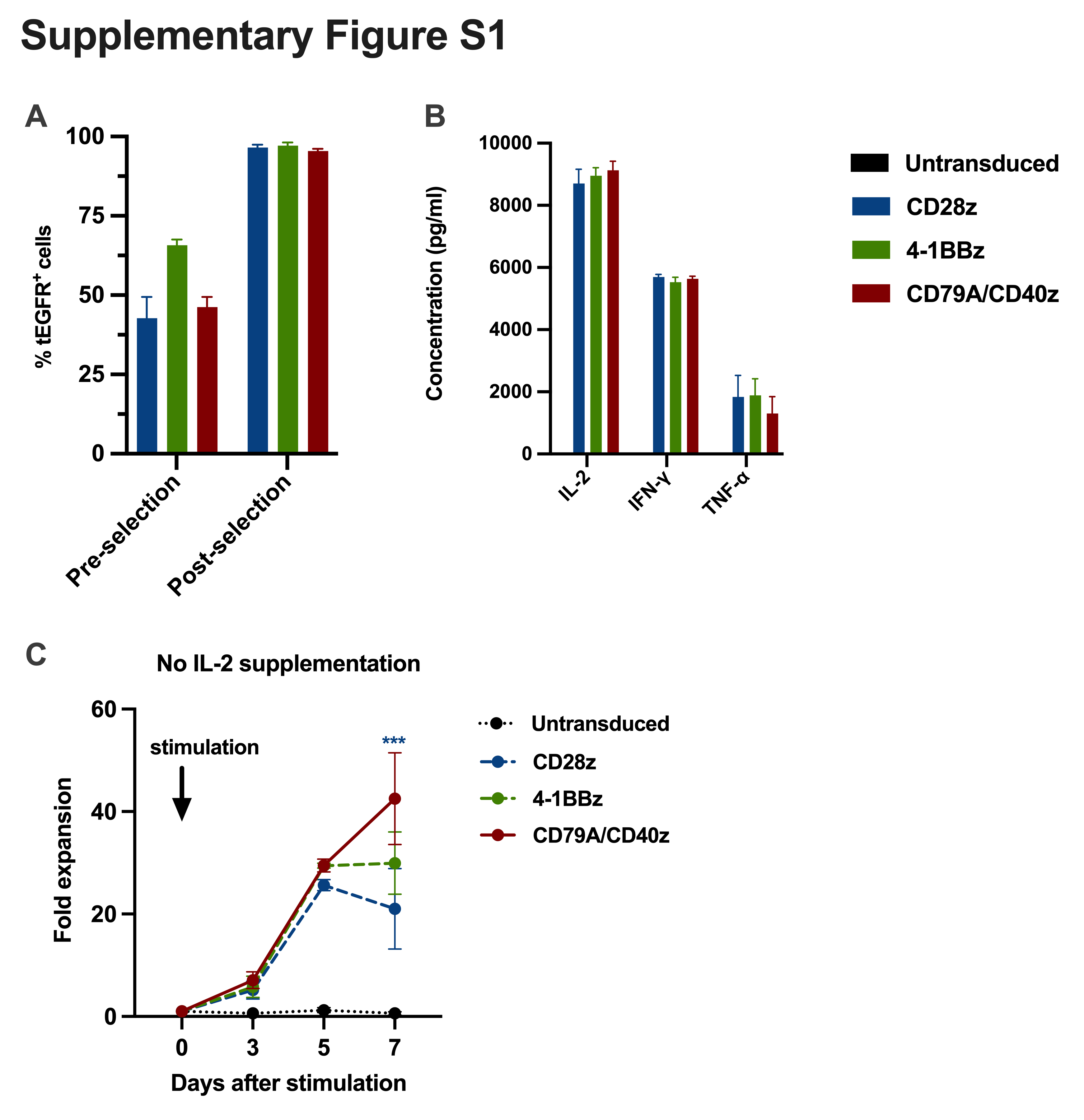

Supplement: Supplementary Figure 1 — (A) Transduction efficacy and purification of CD19CAR-T cells. (B) Concentration of IFN-γ, IL-2, and TNF-α secretion. T-cells were stimulated with γ-irradiated CD19-K562 cell line in a 1:1 ratio for 16 hours, and culture supernatants were analyzed using an ELISA. (C) Fold expansion of T-cells following γ-irradiated CD19-K562 cell line stimulation in a 1:1 ratio for seven days and cultured without IL-2 supplementation. Arrows mark the day of CD19-K562 cell stimulation. All data were pooled from three different donors and are shown as mean ± SEM. One-way ANOVA for (A) and (B); 2-way ANOVA for (C); ***p < 0.001 (CD19.79A.40zCAR- vs. CD19.28z CAR-T cells). [file Image_1.tiff]

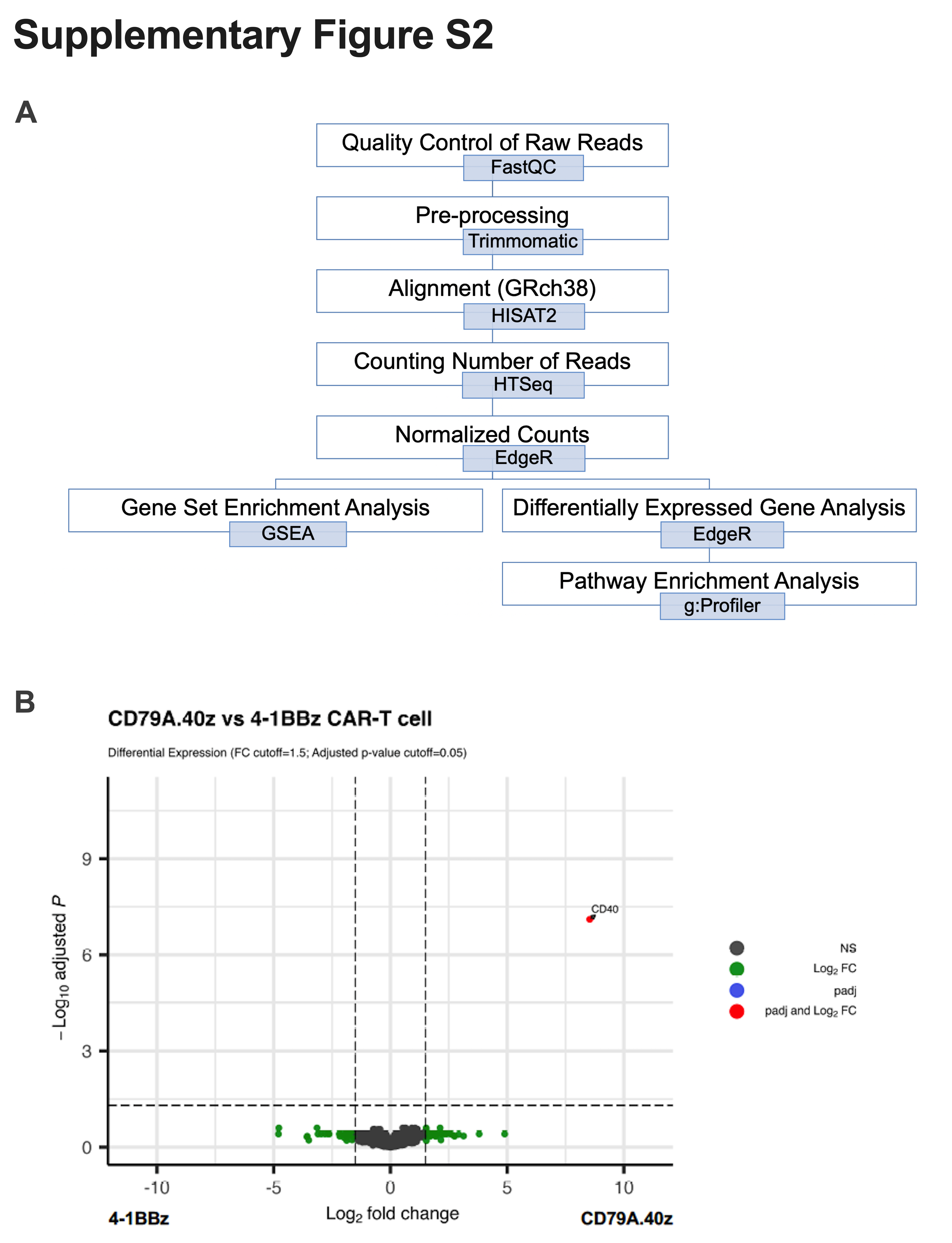

Supplement: Supplementary Figure 2 — (A) RNA-analysis pipeline. (B) Volcano plots of DEGs between CD19.79A.40z CAR-T cells vs. CD19.BBz CAR-T cells. Visualization of DEGs in volcano plots. Up-regulated- and down-regulated genes with FDR < 0.05 are marked in red. DEGs were selected with thresholds of false discovery rate (FDR) ≤ 0.05 and ≥ 1.5-fold difference. [file Image_2.tiff]

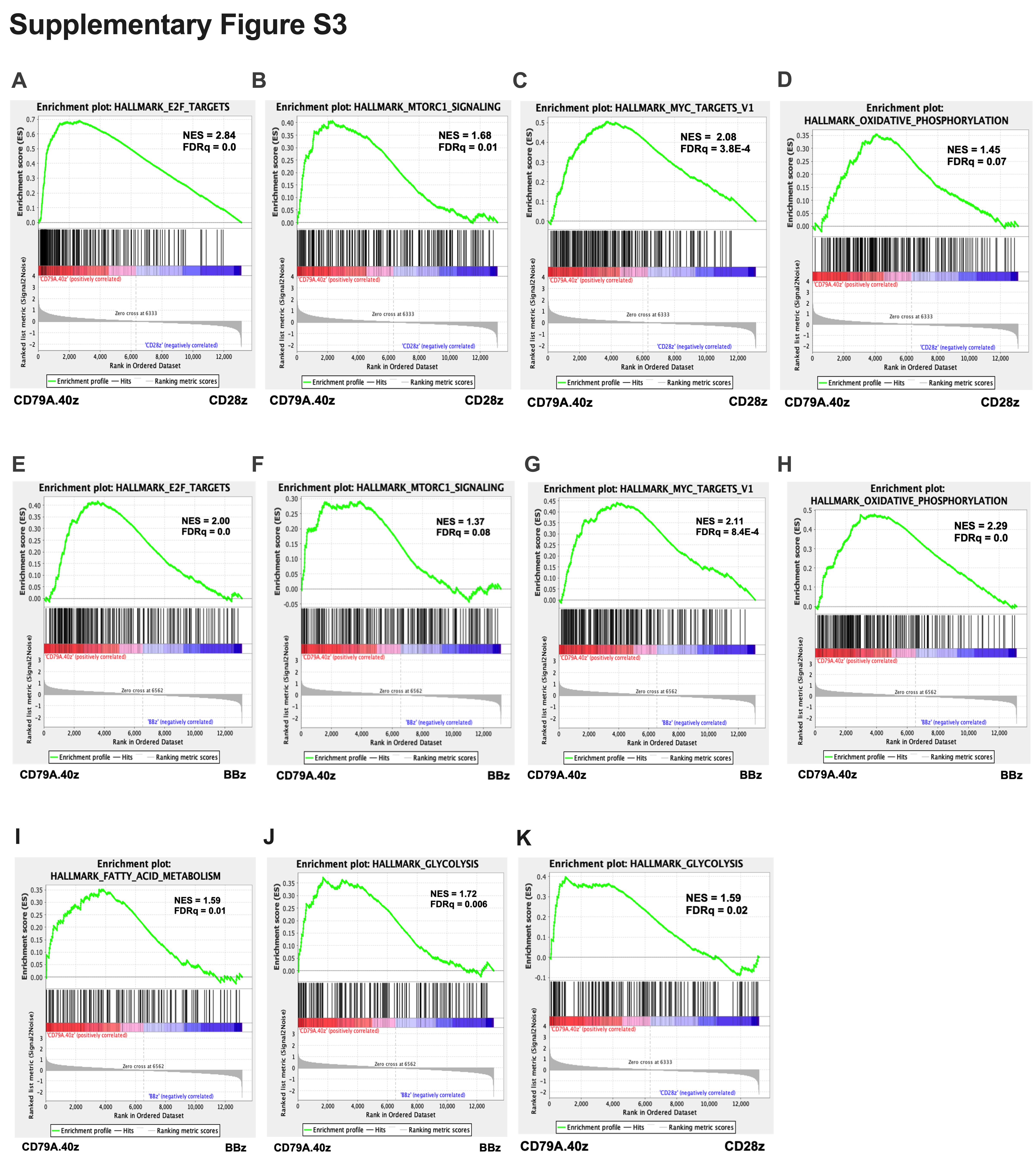

Supplement: Supplementary Figure 3 — (A–D, K) GSEA indicated the gene enrichments in CD19.79A.40z CAR-T cells vs. CD19.28z CAR-T cells in the following hallmarks; (A) E2F target, (B) Mtorc1 signaling, (C) Myc target v1, (D) oxidative phosphorylation, and (K) glycolysis. (E–J) GSEA indicated gene enrichment in CD19.79A.40z CAR-T cells vs. CD19.BBz CAR-T cells in the following hallmarks; (E) E2F target, (F) Mtorc1 signaling, (G) Myc target v1, (H) oxidative phosphorylation, (I) fatty acid metabolism, and (J) glycolysis. The upper part of each plot displays the enrichment score, whereas the lower part displays the ranked list metric of the gene set. The ranked gene list is shown in the middle: red indicates up-regulation; blue indicates down-regulation; and the black vertical line indicates the gene set. FDR q-value cutoff ≤ 0.25, nominal p-value ≤ 0.05. [file Image_3.tiff]
